# Genome assembly of *Ramulariopsis pseudoglycines*, a fungal pathogen responsible for areolate mildew on cotton

**DOI:** 10.1128/mra.01099-24

**Published:** 2025-02-20

**Authors:** Teddy Garcia-Aroca, Robert C. Kemerait, Alejandra M. Jimenez Madrid

**Affiliations:** 1 Department of Plant Pathology, University of Nebraska-Lincoln, Lincoln, Nebraska, USA; 2 Department of Plant Pathology, University of Georgia, Tifton, Georgia, USA; University of Guelph, Guelph, Ontario, Canada

**Keywords:** genomes, plant pathogens, cotton, fungal pathogen, nanopore, whole genome sequencing

## Abstract

The fungal species *Ramulariopsis pseudoglycines* was recently reported as a pathogen of cotton in the United States. Here, we report the first genome of this pathogen assembled using nanopore sequencing. The genome size is ~77.9 Mb, with an *N*_50_ of ~2.5 Mb, GC content of 52.74%, and Benchmarking Universal Single-Copy Orthologs (BUSCO) completeness of 99.5%.

## ANNOUNCEMENT

The causal agent of areolate mildew of cotton (*Gossypium hirsutum* L.) was originally described as *Ramularia areola* (1), but the species was reclassified to *Ramulariopsis gossypii* (2). Multi-locus DNA sequencing revealed that *Ramularia pseudoglycines* is also associated with this disease (3). Currently, *R. gossypii* and *R. pseudoglycines* are both responsible for areolate mildew, and the latter is the most widespread species in Brazil affecting yield and fiber quality (4, 5). *R. pseudoglycines* was reported in the United States for the first time in 2023 (6), and it is becoming prevalent in the Southeastern United States. Given its importance, a genome assembly is needed to further investigate its pathogenicity, host-pathogen interactions, and ecology.

In August 2023, commercial cotton plants in Georgia (Colquitt County) showed yellow lesions in the adaxial leaf surface and white powdery fungal sporulation in the abaxial surface. Conidia observed from the leaf surface was scraped with a sterile scalpel onto V8 medium amended with chloramphenicol (75 ppm) and streptomycin sulfate (125 ppm) and incubated in the dark at 25°C. Fungal colonies developed after 6 days, and they were consistent with previous *Ramulariopsis* spp. descriptions (3, 6, 7). These colonies were transferred to V8-antibiotic-amended medium to obtain pure cultures. Isolate AJ029-2023 (culture collection: FELCC 511) was selected for whole-genome sequencing. Single colonies were restored from 30% glycerol stocks, and fungal biomass was grown in liquid V8 media at room temperature under shaking conditions (150 rpm) for 10 days.

High molecular weight genomic DNA was isolated using a modified CTAB protocol (8) and adjusted to 30 ng µL^−1^ in nuclease-free water. Genomic DNA was prepared for sequencing by first applying a DNA repair and end-prep step, then adapter ligation and clean up using the Ligation Sequencing Kit V14 (SQK-LSK114) without shearing or size selection, as specified in the manufacturer’s protocols. DNA was sequenced using the Oxford Nanopore MinION Mk1C platform and the R10.4.1 Flow Cell (FLO-MIN114). Sequencing quality was monitored using the MinKNOW version 22.10.5 GUI interface. Sequences were base called and demultiplexed using Guppy version 6.3.8 (9). The total number of reads was 12.71 million, and the estimated read *N*_50_ was 3.36 kb. The genome was assembled with Flye version 2.9.1 (10) and polished with Medaka version 1.7.2 (11), using a *de novo* assembly approach. Assembly quality was assessed with Quast version 5.0.2 (12) and completeness with the Benchmarking Universal Single-Copy Orthologs (BUSCO) software version 5.4.3 (13). Gene prediction and annotation were performed with the COMprehensive Parasite ANnotatION version 2.2.8 database (14), using *Zymoseptoria tritici* IPO323 version 2.0 (15, 16) as a reference. Default parameters were used for all software unless otherwise speciﬁed. The genome size is 77,925,435 bp, with 148 contigs, *N*_50_ of 2,570,697 bp, GC content of 52.74%, and BUSCO completeness (ascomycota_odb10) of 99.5% (Table 1).

**TABLE 1:** Genome sequencing, assembly statistics, and genomic features of *Ramulariopsis pseudoglycines* isolate AJ029-2023 (FELCC 511)

Sequencing/genomic features	
No. of reads	12.71 million
Bases called	23.43 Gb
Contigs	148
Longest contig	8,117,343 bp
Genome size	77,925,435 bp
*N*_50_	2,570,697 bp
GC content	52.74%
BUSCO completeness (ascomycota_odb10)	99.5%
CDS	76,268
Coding genes	24,936
Ribosomal RNAs	63
Transfer RNAs	141

## Data Availability

The information associated with this genome can be found at NCBI BioProject PRJNA1162073 and BioSample SAMN43800085. Raw sequence reads were submitted to the NCBI Sequence Read Archive (SRA) SRR30690797. This Whole Genome Shotgun project has been deposited at GenBank under the accession JBHJNK000000000. The version described in this paper is GenBank GCA_043513825.1.
